# Cytologic findings in malignant myoepithelioma: a case report and review of the literature

**DOI:** 10.1186/1742-6413-4-3

**Published:** 2007-01-25

**Authors:** Torill Sauer

**Affiliations:** 1 Department of Pathology, Faculty Division Ullevaal University Hospital, N-0407 Oslo, Norway

## Abstract

**Background:**

Myoepithelioma of the breast is a rare tumor and the cytologic features have only been described in one previous report.

**Case presentation:**

The present case comprises a 70 year old woman with a mammographic equivocal and ultrasonographic suspicious lesion. The aspirates were cellular and consisted mainly of single spindle or polymorphic, polygonal cells. The nuclei were generally large, ranging from 2 - > 5 × RBC. Most nuclei had a distinct medium-sized nucleolus. The nuclear outlines were irregular with buds and folds. The chromatin was granular. In the background there was abundant granular metachromatic ground substance and some metachromatic stromal fragments. A few mitotic figures were found. The cytologic diagnosis was suspicious for malignancy and a metaplastic carcinoma where only the non-epithelial component had been aspirated, or a non-epithelial lesion, was suggested.

Macroscopically the tumor was round, seemingly well circumscribed, firm and with a white cut surface. The lesion consisted of spindled and polygonal cells with distinct pleomorphism. There were 6–9 mitoses per high power field (HPF). The tumor infiltrated in the surrounding fatty tissue. On immunohistochemistry, tumor cells were positive for smooth muscle actin, keratin MNF 116 and vimentin. Desmin and S-100 were negative.

Ultrastructurally, there were abundant tonofilaments, including globular filamentous bodies and granulated endocytoplasmic reticulum with many dilated cisterns. The histologic diagnosis was malignant myoepithelioma.

**Conclusion:**

The case mirrors completely the WHO definition and the previous cytological and histological descriptions of malignant myoepitheliomas in the literature which describe a spindle cell population with unequivocal nuclear atypia, metachromatic background substance and mitoses.

## Background

Myoepithelioma of the breast is a rare tumor. It usually presents as a palpable nodule, and a mammographic density without distinctive features. Most are benign, and only few malignant cases have been reported in the literature [1-4]. The patient age may range from 22 to 87 years. WHO Classification of Tumours: "Tumours of the Breast and Female genital Organs" [5] define malignant myoepithelioma as an infiltrating tumour composed purely of myoepithelial cells (predominantly spindled) with identifiable mitotic activity. They may vary in size from 1 cm to 21 cm. The cytologic findings in a malignant myoepithelioma have been described in one previous report [6]. This paper describes the findings in an additional case. The findings are compared with the characteristic features of histologic findings that are described in the literature.

## Case presentation

### Clinical history

A 70-year old woman attended mammography screening. The mammograms revealed en equivocal lesion. Ultrasonography identified a 14 mm tumor that was suspicious for malignancy. A FNAC was done under ultrasound guidance.

### FNAC findings

**Figure 1:**
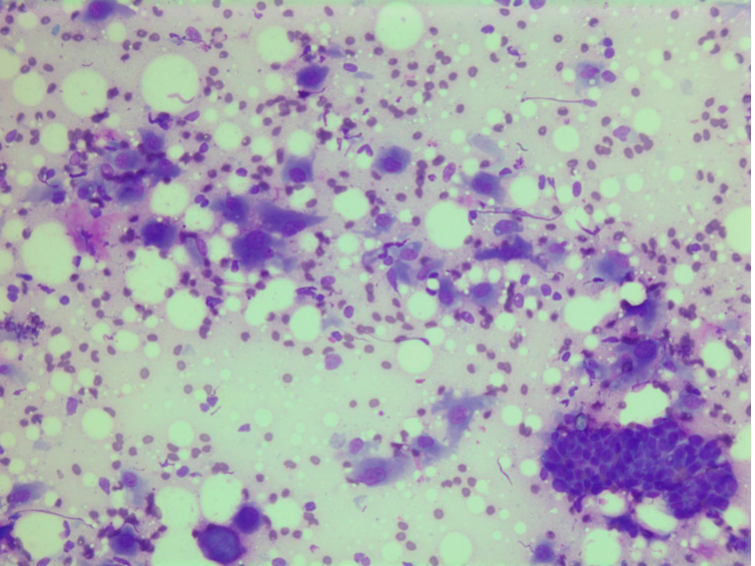
Air dried, Giemsa stained smear showing single spindle or polymorphic, polygonal cells with and group of benign ductal epithelial cells.

The smears were stained with Diff-Quick^® ^(Dade AG, Düdingen, Germany). The aspirates were cellular and consisted mainly of single spindle or polymorphic, polygonal cells with a few admixed groups of benign ductal epithelial cells (Figure 1) and lymphocytes.

**Figure 2:**
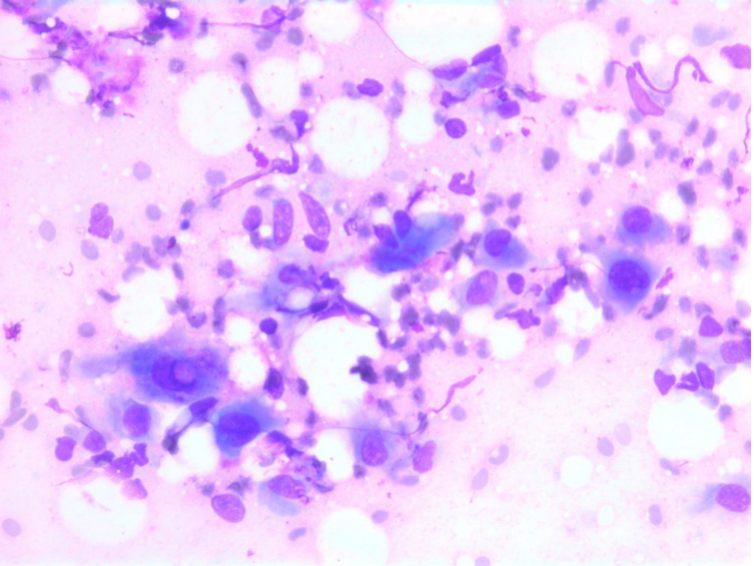
Air dried, Giemsa stained smear showing a mixture of spindle and polygonal cells in a granular metachromatic ground substance. One of the polygonal cells has an intranuclear vacuole.

**Figure 3:**
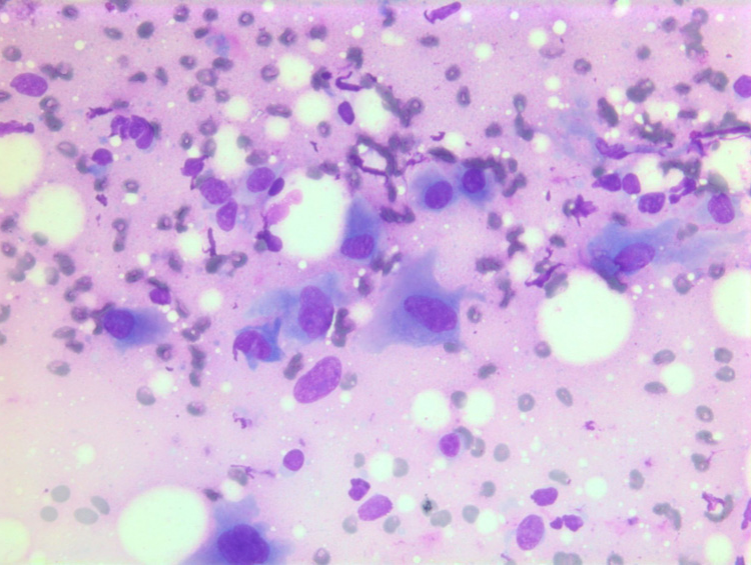
Air dried, Giemsa stained smear with polygonal tumor cells with dense bluish cytoplasm.

**Figure 4:**
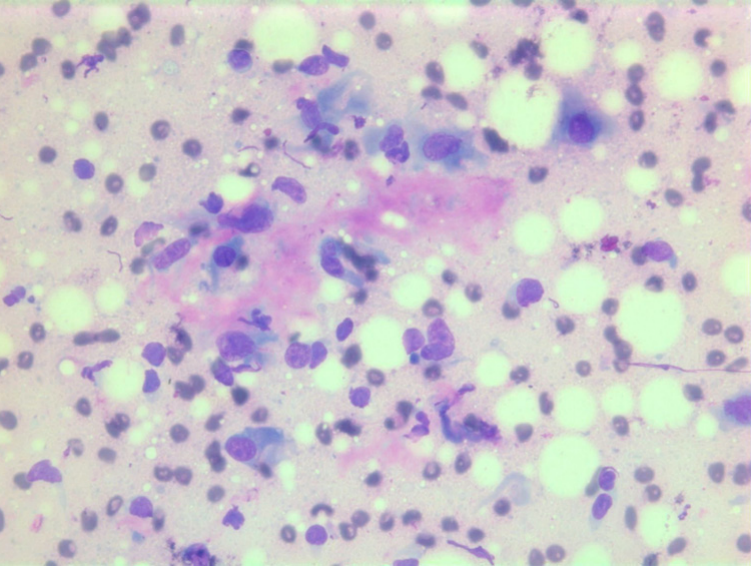
Air-dried Giemsa stained smear showing abundant granular metachromatic ground substance and a metachromatic stromal fragment.

**Figure 5:**
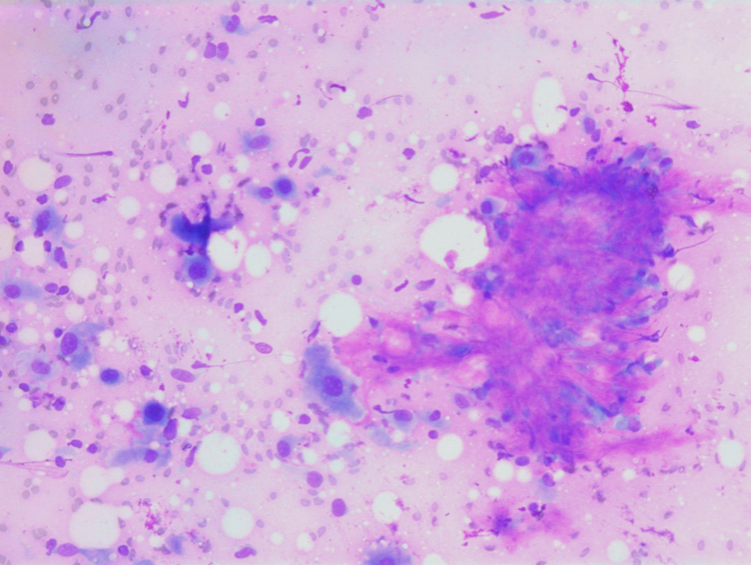
Air-dried Giemsa stained smear with large metachromatic stromal fragment.

**Figure 6:**
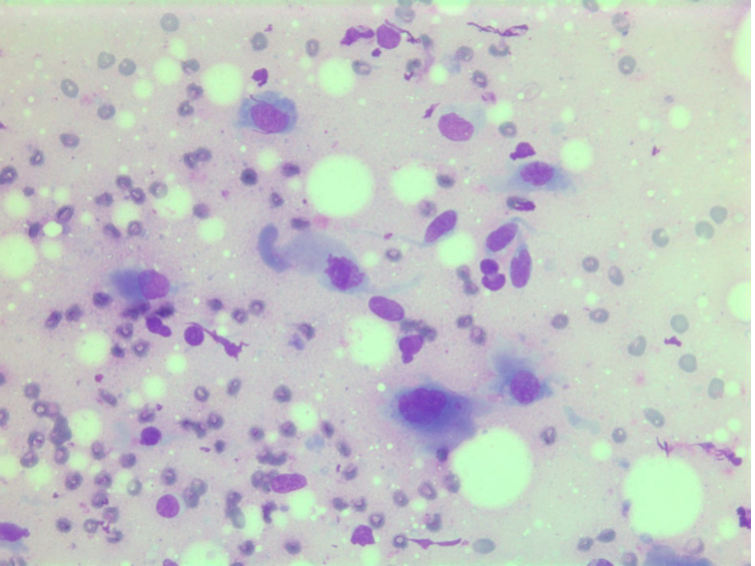
Air-dried Giemsa stained smear central mitotic figure (in the center of the image).

The nuclei were generally large, ranging from 2 - > 5 × RBC. Most nuclei had a distinct medium-sized nucleolus. The nuclear outlines were irregular with buds and folds. The chromatin was granular. A few cells showing intranuclear cytoplasmic vacuoles were found (Figure 2). The cytoplasm was bluish, variable in amount and often dense (Figure 3). In the background there was abundant granular metachromatic ground substance and some metachromatic stromal fragments (Figures 2, 4 and 5). A few mitotic figures were found (Figure 6). There was no necrotic debris.

The cytologic diagnosis was suspicious for malignancy and a metaplastic carcinoma where only the non-epithelial component had been aspirated, or a non-epithelial lesion, was suggested. No smears were available for immunocytochemistry.

### Histopathological findings

**Figure 7:**
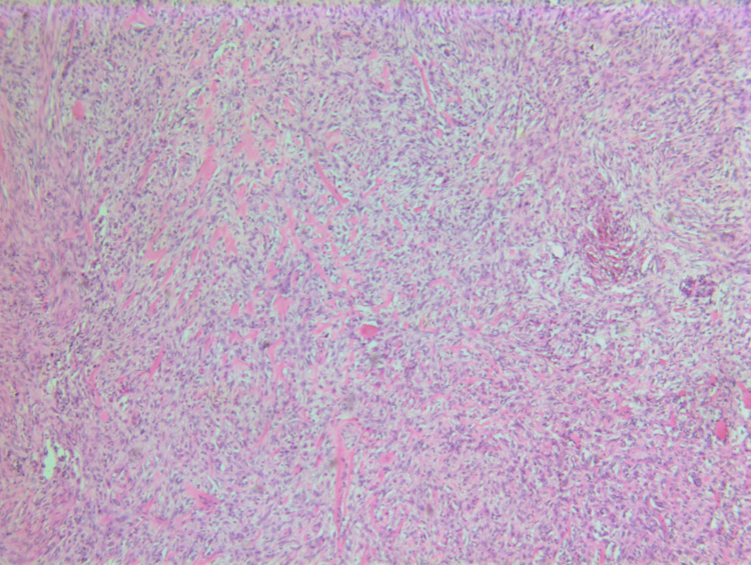
HE stain. Histopathological specimen showing a cell dense tumor with eosinophilic ground substance.

**Figure 8:**
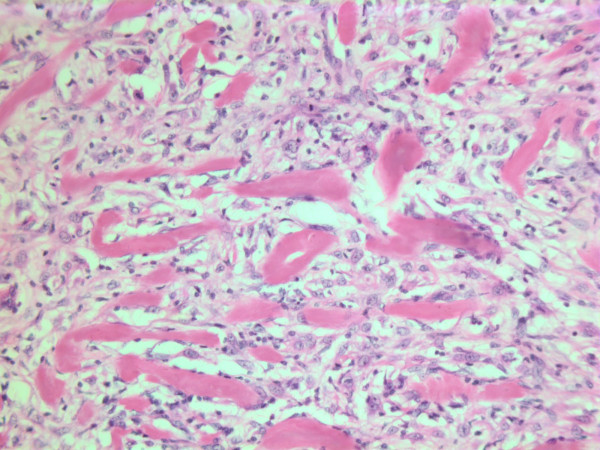
HE stain. Histopathological section of the tumor with a dense infiltrate of atypical cells and an eosinophilic substance in between.

**Figure 9:**
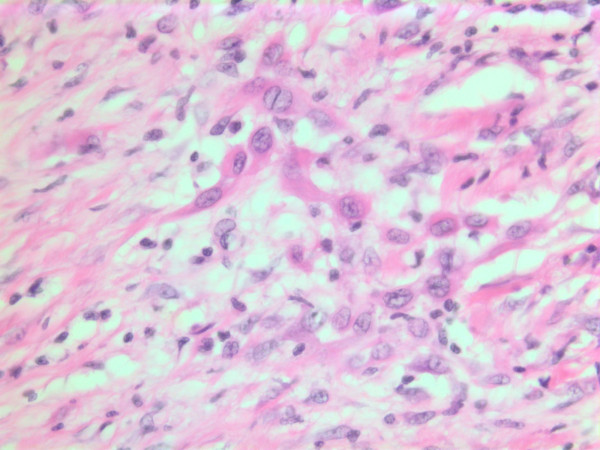
HE stain. Histological section of the tumor with spindled and polygonal cells with distinct pleomorphism and dense eosinophilic cytoplasm.

**Figure 10:**
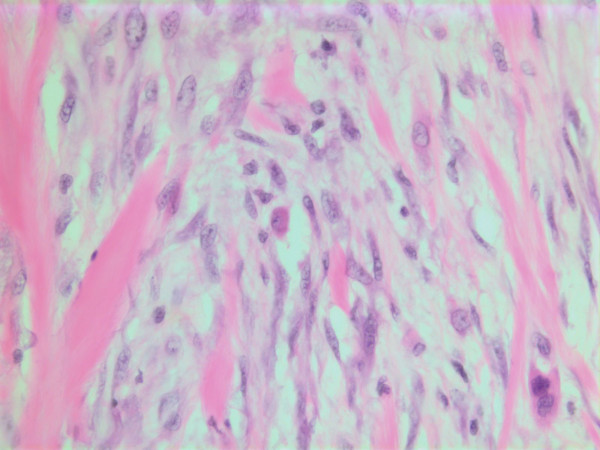
HE stain. Histological section of the tumor with mainly spindled tumor cells and a dense eosinophilic intercellular substance.

**Figure 11:**
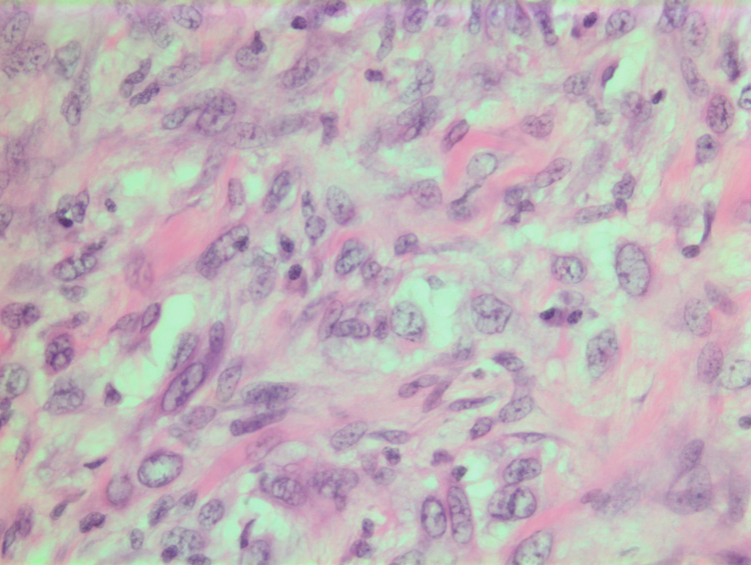
HE stain. Histological section of the tumor with spindled and polygonal tumor cells with distinct pleomorphism.

**Figure 12:**
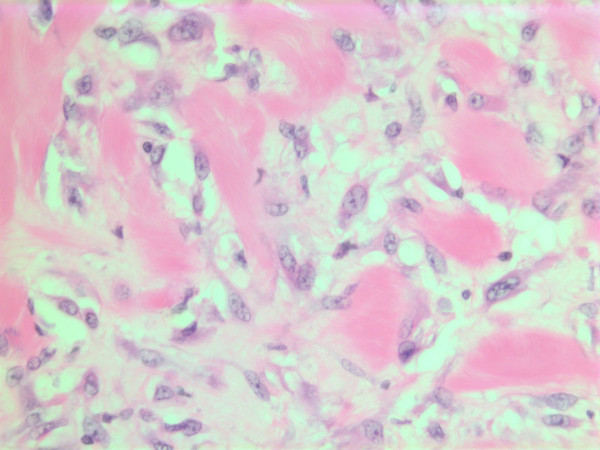
HE stain. Histological section of the tumor showing an acellular eosinophilic ground substance with pleomorphic tumor cell population.

**Figure 13:**
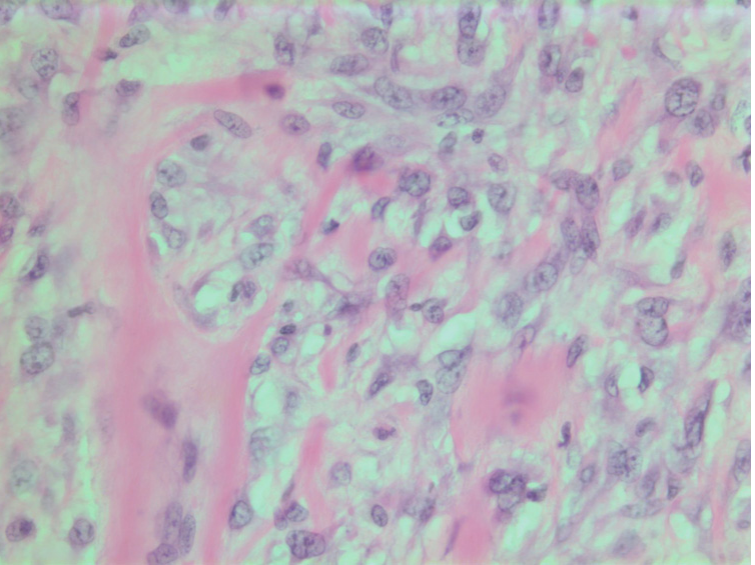
HE stain. Histopathological section from tumor area with a dense tumor cell infiltrate and some eosinophilic substance.

**Figure 14:**
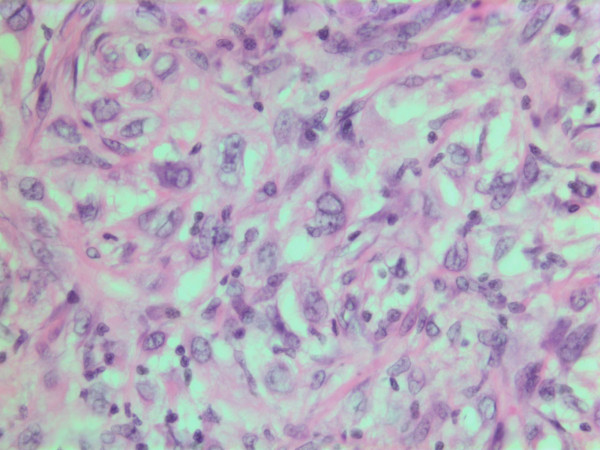
HE stain. Admixture of lymphocytes in histological section of the tumor.

**Figure 15:**
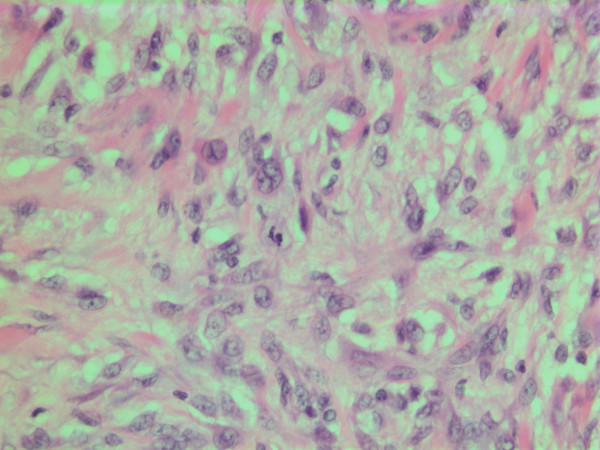
HE stain. Histological section of the tumor showing pleomorphic tumor cells with central mitotic figure.

**Figure 16:**
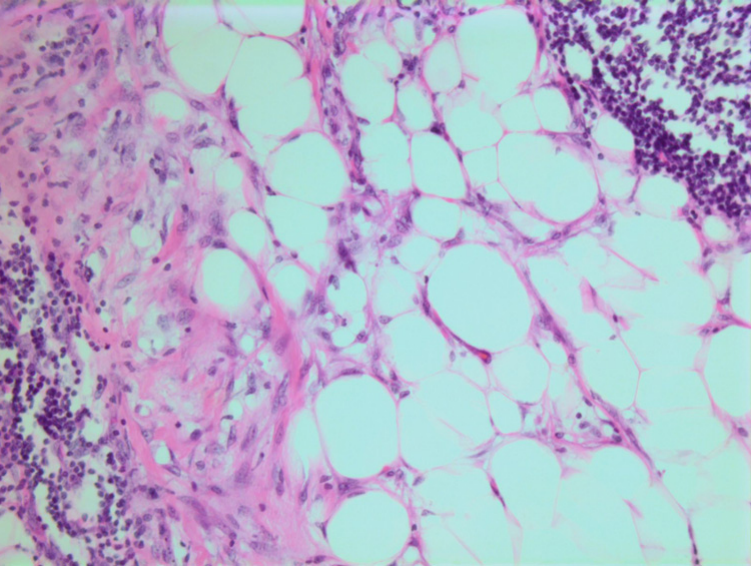
HE stain. Histological section from area demonstrating tumor cell invasion in surrounding fatty tissue.

**Figure 17:**
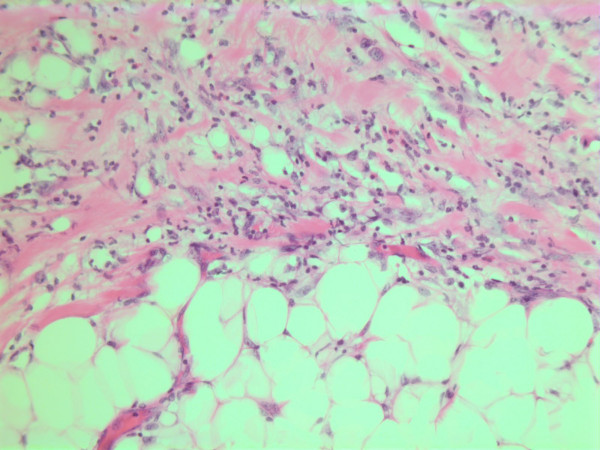
HE stain. Histological section from the tumor with fuzzy invasion border towards the fatty tissue.

**Figure 18:**
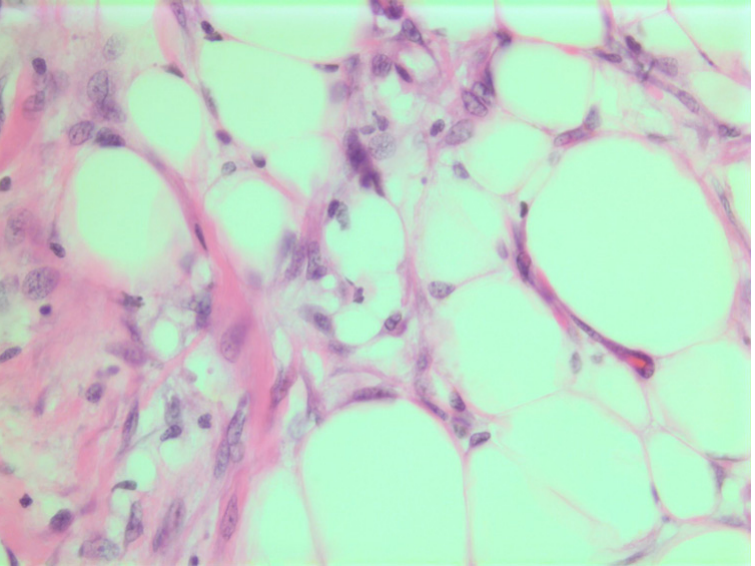
HE stain. Histological section of the tumor demonstrating invasion of spindled tumor cells in fatty tissue.

The histopathological characteristics are shown in Figures 7, 8, 9, 10, 11, 12, 13, 14, 15, 16, 17, 18. Macroscopically the tumor was round, seemingly well circumscribed, firm and with a white cut surface. The diameter was 14 mm. On microscopy, the lesion was cellular (Figures 7 and 8), consisting of spindled and polygonal cells with distinct pleomorphism (Figures 9, 10, 11). There were variable amounts of eosinophilic ground substance (Figures 8, 12 and 13) and a focal admixture of lymphocytes (Figures 9 and 14). There were 6–9 mitoses per high power field (HPF) (Figure 15) The tumor infiltrated in the surrounding fatty tissue (Figures 16, 17, 18).

**Figure 19:**
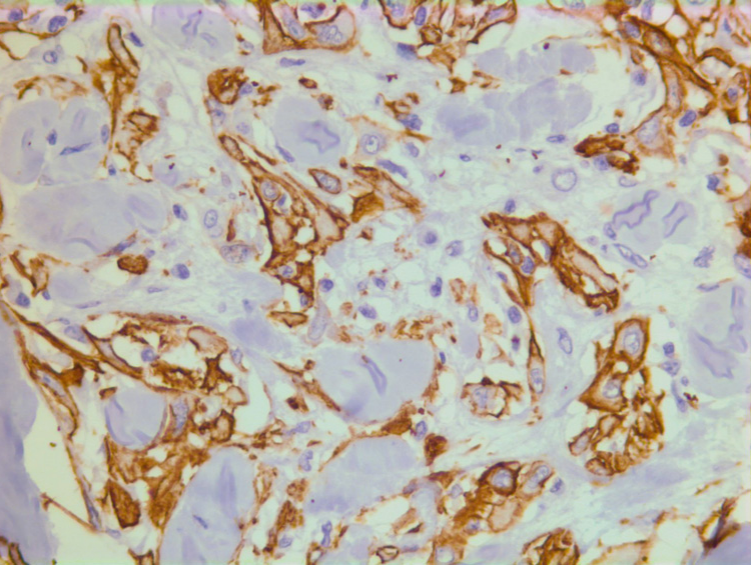
Immunohistochemical stain for actin which is positive in the tumor cells.

**Figure 20:**
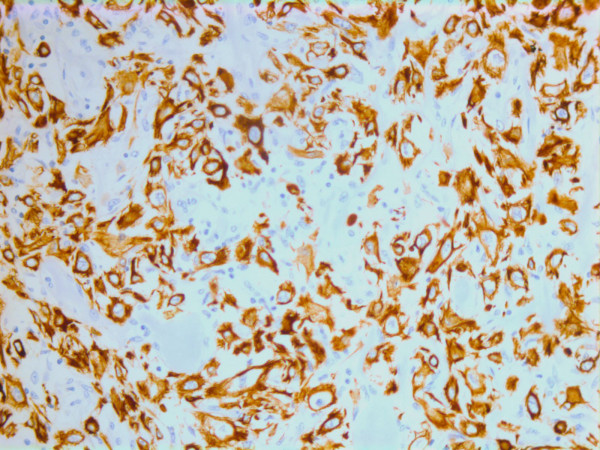
Immunohistochemical stain with a pan-keratin AB.

**Figure 21:**
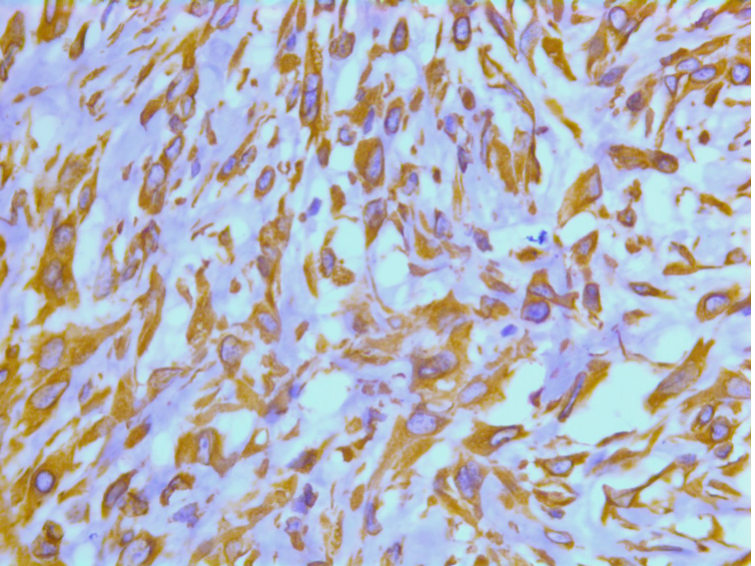
Immunohistochemical stain with a vimentin AB.

**Figure 22:**
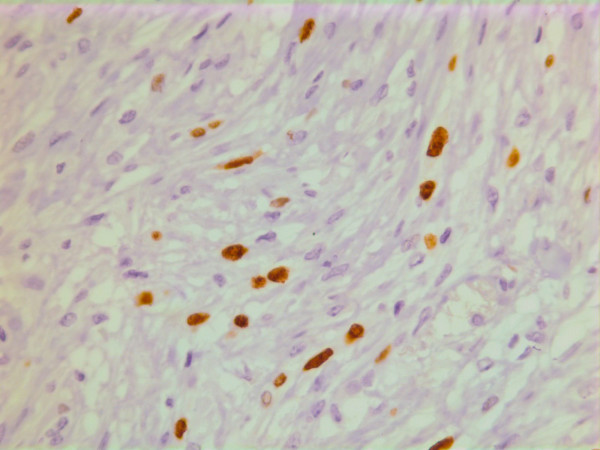
Immunohistochemical stain with the proliferation marker Ki-67.

On immunohistochemistry, the tumor cells were positive for smooth muscle actin (DAKO, Glostrup, Denmark) (Figure 19), keratin MNF 116 (a pan-keratin with both high- and low molecular weight keratins from DAKO, Glostrup, Denmark) (Figure 20) and vimentin (DAKO, Glostrup, Denmark)(Figure 21). About 30 % of the tumor cell nuclei were positive for Ki-67 (DAKO, Glostrup, Denmark) (Figure 22). Desmin (DAKO, Glostrup, Denmark) and S-100 (DAKO, Glostrup, Denmark) were negative. Estrogen- and progesterone receptors (DAKO, Glostrup, Denmark) as well as HER-2 (Novocastra, Newcastle upon Tyne, UK) were all negative.

**Figure 23:**
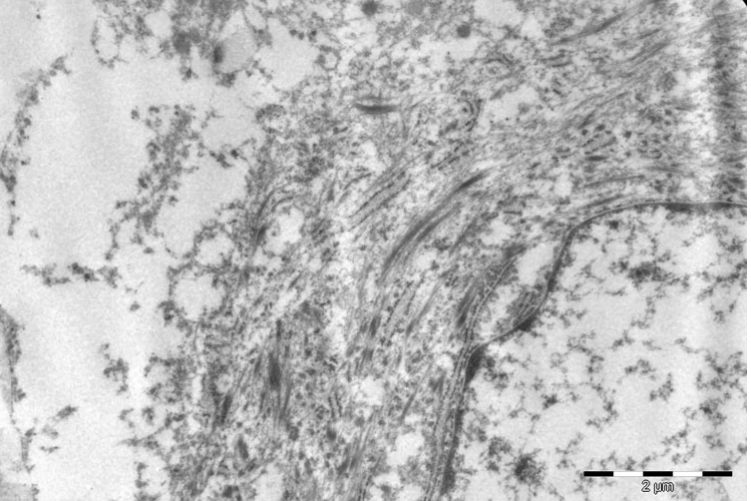
Ultrastructural image showing abundant tonofilaments in the cytoplasm.

Ultrastructurally, there were abundant tonofilaments (Figure 23), including globular filamentous bodies and granulated endocytoplasmic reticulum with many dilated cisterna. Desmosomes were not identified, but the tissue was poorly preserved. A few lysosomes were seen.

The histologic diagnosis was malignant myoepithelioma. The sentinel axillary lymph node was examined and was negative.

## Discussion

The cytologic features in this case presented solely with mesenchymal appearing cells with a distinct nuclear atypia. This is in keeping with previous histologic reports that malignant myoepitheliomas usually present with a spindle cell population and no epithelial cell component [1,2,4]. Kurashini et al [6] reported a case of malignant myoepithelioma consisting mainly of spindle-shaped cells with a few admixed epithelial cells. They reported nuclear atypia with hyperchromasia and prominent nucleoli, occasional intranuclear cytoplasmic inclusions and mitoses. Our findings are practically identical. Kurashina et al did not comment upon the ground-/background substance, which probably was difficult to appreciate in their Papanicolaou stained smears where it is well known that extracellular material might be difficult or impossible to see.

The cytological criteria of benign myoepitheliomas of the breast are known [7-12]. They present with a dual cell population with clusters of both epithelial and spindled cells. The cells may show a mild to moderate nuclear pleomorphism, occasional intranuclear cytoplasmic vacuoles, naked bipolar cells and a metachromatic, fibrillary, myxoid material, but no necrosis or mitoses.

The cytological criteria of malignant myoepitheliomas have not been established, but the findings are concurrent in the case of Kurashini et al [6] and our case. Both cases mirror completely the WHO definition [5] and the histological descriptions of malignant myoepitheliomas in the literature [1-4] which describe a spindle cell population with unequivocal nuclear atypia, metachromatic background substance and mitoses.

The cytologic differential diagnoses of malignant spindle cell tumors include borderline and malignant phyllodes tumor, soft tissue sarcomas as leiomyosarcoma, fibrosarcoma, malignant fibrous histiocytoma and malignant schwannoma. Immunocytochemical stains may be of help when additional smears, cell suspensions or cell blocks are available. A suitable panel of markers would include antibodies against epithelial and myoepithelial differentiation, mesenchymal and smooth muscle as well as neural differentiation. Positivity for epithelial and myoepithelial markers would rule out phyllodes tumor and all types of soft tissue sarcomas.

Another important differential diagnosis is metaplastic carcinoma. In most cases of FNAC from metaplastic carcinomas there will be a distinct carcinomatous component. A few cases may present only the mesenchymal appearing component on FNAC. In contrast to malignant myoepithelioma, the mesenchymal component in metaplastic carcinomas will express epithelial markers, at least focally with the exception of rare true carcinosarcomas. The combined epithelial/myoepithelial immunophenotype may be found in some metaplastic carcinomas also. A preoperative, cytologic and immunocytologically distinction between a malignant myoepithelioma and a metaplastic carcinoma where only the mesenchymal appearing component is present might not always be possible.

## Conclusion

This case mirrors completely the WHO definition and the previous cytological and histological descriptions of malignant myoepitheliomas in the literature which describe a spindle cell population with unequivocal nuclear atypia, metachromatic background substance and mitoses. Immunocytochemistry might aid in narrowing the differential diagnoses, but a specific cytologic diagnosis might still not be possible.

## Abbreviations

HPF = high power field

RBC = red blood cell

WHO = world health organization

FNAC = fine needle aspiration cytology

## Conflicts of interest

There are no conflicts of interest

This case report is the sole work of the author Torill Sauer.
